# Stakeholder Engagement Behavior(s) in Sustainable Brownfield Regeneration: A Network Embeddedness Perspective

**DOI:** 10.3390/ijerph19106029

**Published:** 2022-05-16

**Authors:** Hongli Lin, Yuming Zhu, Jiahe Zhou, Bingxu Mu, Caihong Liu

**Affiliations:** School of Management, Northwestern Polytechnical University, Xi’an 710100, China; lhl2016@mail.nwpu.edu.cn (H.L.); bingxu0908@mail.nwpu.edu.cn (B.M.); caihongliu@mail.nwpu.edu.cn (C.L.)

**Keywords:** brownfield regeneration, relationship network structure, engagement behavior, influence mechanisms

## Abstract

Brownfield regeneration (BR) is an important initiative for sustainable land development and the promotion of carbon neutrality. Insufficient stakeholder engagement is one of the main obstacles to the progress of BR. The relationship network formed through continuous interaction among stakeholders ensures the exchange and transfer of information resources. Different structural features of the relationship network may lead to differences in the engagement level of stakeholders. Therefore, based on network embeddedness theory, this study conducts an empirical analysis to explore the impact of the relationship network structure on engagement behavior, for the purpose of increasing the stakeholder engagement level. A theoretical model is developed, in which network centrality, behavioral willingness and involvement climate are selected as the dependent variables of engagement behavior. Using an effective sample of 245 stakeholders involved in BR from ten cities in China, we find that stakeholder network centrality positively affects engagement behavior. There is also a positive relationship between network centrality and behavioral willingness. The behavioral willingness plays an intermediary role between network centrality and engagement behavior, and the involvement climate has a moderating role between behavioral willingness and engagement behavior. Additionally, various strategies can be adopted to promote the engagement level of stakeholders. The findings are useful in establishing a benchmarking framework for BR stakeholder engagement.

## 1. Introduction

Brownfield sites, defined as “any land or premises which has previously been used or developed and is not currently fully in use, although it may be partially occupied or utilized, and it may also be vacant, derelict, or contaminated” [1], represent an opportunity for urban development. BR has inherent sustainability and is of great significance in the sustainability of urban development [2,3,4,5,6] and land management [7]. In the land management policy of China, soil pollution control and remediation are regarded as outstanding environmental problems to be solved. In the context of optimizing the spatial pattern of national land, BR can convert brownfield sites into ecological land, which is an important measure for reducing carbon emissions and enhancing the carbon sink capacity of the ecosystem, and for achieving the strategic goal of “2030 carbon peak, 2060 carbon neutral” in China [8]. The importance and urgency of BR are increasingly highlighted to preserve the value of land assets and achieve the goal of carbon neutrality as soon as possible.

Sustainable BR refers to remediation and management in an environmentally sensitive, economically feasible, institutionally robust and socially widely acceptable way that continues to meet the needs of present and future generations [9]. Like all public issues with a long period and a wide range of impacts, BR also involves a wide range of stakeholders, which has become a key requirement of sustainable development strategies [10]. When more stakeholders are involved in BR, more stakeholder engagement issues occur [11]. For example, inactive engagement behavior may result in group incidents, which is one of the barriers to the achievement of sustainable BR. The engagement behavior of stakeholders is defined as the manifestation of behavior motivated by the integrative effects of emotion and cognition [12]. Hollebeek et al. [13] considered stakeholders’ engagement behavior as the resource endowment behavior that is formed in their role-related interactions, activities or relationships, which are bounded and voluntary. Combining the studies of Viglia et al. [12] and Hollebeek et al. [13], this paper defines stakeholder engagement behavior as the degree of behavioral activism resulting from the interaction of different stakeholders in BR.

A growing number of scholars have recognized the importance of stakeholders’ engagement behaviors for the success of marine spatial planning, the effectiveness of estuary environmental governance, the success of value co-creation in project management, urban energy planning development, the sustainability of corporate environmental policies, successful freshwater resource management and the robustness of complex environmental governance processes [14,15,16,17,18,19,20]. More importantly, more and more scholars have recognized the importance of stakeholder engagement in BR and explored it from different perspectives. Hou et al. [21] used structural equation models to analyze the effects of stakeholder engagement on the adoption of sustainable regeneration practices. Bayulken and Huisingh [22] analyzed stakeholder engagement sustainability from the perspective of engagement breadth and depth in Nordic BR. Alexandrescu et al. [23] found that the social embeddedness of stakeholders affected the degree and effectiveness of engagement.

Based on the complex social person hypothesis of stakeholders, stakeholder engagement behavior is influenced by their social attributes. As in user engagement, interaction is at the heart of stakeholder engagement. This interaction is defined as face-to-face or other forms of interaction between two or more stakeholders [24,25]. In the process of stakeholder interaction, Jahangirian et al. [26] found that the communication relationship between stakeholders influenced the level of stakeholder engagement in healthcare projects. Ju and Wei [27] studied the impact of user social networks on the level of user engagement in ERP systems. Based on a qualitative case study, Butt et al. [28] explored how communication relationships influenced the engagement behavior of stakeholders in the project change management process. Different stakeholders have different social relationships, and these social relationships have an impact on stakeholder engagement [13]. These studies indicate that the relationships between stakeholders influence their engagement from the qualitative perspective, without considering the social embeddedness of stakeholders, such as the relationship network structure. In the process of stakeholder engagement, there are intricate relationships among them, which naturally form the relationship network. To achieve the reality and the desired level of stakeholder engagement, the influence of relationship network structure on engagement behavior is worth considering.

There is a trend towards applying social network analysis (SNA) to study the stakeholder relationships in many large and complex public projects [29,30,31,32]. However, studies on the correlation between network structure and engagement behavior have not yet been presented. As an important component of sustainable BR, the stakeholder relationship network carries the contract, communication, information exchange and trust relationships between different stakeholders. To what extent do different structural characteristics of the relationship network (network centrality) influence stakeholder participation behavior? How do effective mechanisms of stakeholder engagement develop under the effect of relationship network structure, behavioral willingness and involvement climate? These are realistic questions that must be answered in order to achieve the sustainability of BR from the social aspect. Therefore, based on network embeddedness theory, the theory of planned behavior and attitude–context–behavior theory, this paper systematically studies the influence mechanism of the relationship network structure on engagement behavior through empirical analysis, to provide scientific theoretical support for improving the engagement behavior of BR stakeholders.

**Figure 1:**
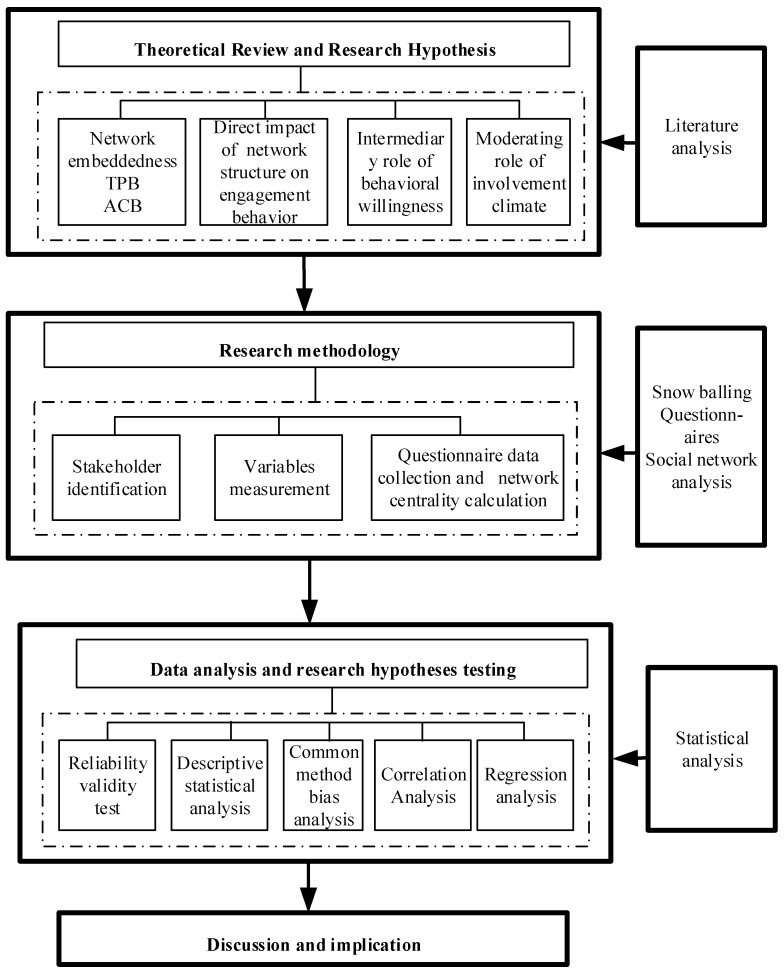
A framework for analyzing the influence of relationship network structure on engagement behavior.

As shown in Figure 1, in this paper, we adopt a literature analysis, a questionnaire survey, correlation analysis and regression analysis to carry out theoretical model construction and research design and testing, and we discuss the impact of the stakeholder relationship network structure on engagement behavior.

Following the introduction, Section 2 discusses social network theory, the theory of planned behavior, attitude–context–behavior theory and the influence hypothesis of the stakeholder relationship network structure on engagement behavior. Section 3 presents the stakeholder identification, variables measurement, questionnaire data collection and network centrality calculation. Section 4 introduces the results of the correlation analysis and regression analysis. Section 5 analyzes the influence mechanism of the stakeholder relationship network structure on engagement behavior and proposes suggestions for improving the motivation of stakeholder engagement behavior. Section 6 summarizes the conclusions and provides future directions.

## 2. Theoretical Review and Research Hypothesis

### 2.1. Theoretical Review

#### 2.1.1. Network Embeddedness Theory

The classical analytical framework of network embeddedness theory includes structure embeddedness and relationship embeddedness. Structure embeddedness theory, as a further explanation of the interplay between economic behavior and social relationships, was developed by Granovetter [33]. It pointed out that economic behavior is embedded in complex social relationships and can be influenced by numerous non-economic factors. Economic behavior is subject to the mechanisms of trust and communication between social actors. These mechanisms are derived from the relationship networks of social actors, and therefore economic behavior is naturally embedded in the trust and communication relationship structure. Structure embeddedness theory analyzes how the size and density of the overall network, the location of social actors in the social network and the network groups formed influence the occurrence of economic behavior. Structural embeddedness can help individuals in the network to access knowledge and information resources that are lacking within the organization, expand knowledge boundaries and enhance their behavioral ability to cope with radical change [34]. Li et al. [35] argued that heterogeneous resources acquired by employees through structural embeddedness have become a key element in promoting innovative behavior. Belso-Martinez et al. [36] suggested that employees are able to access more information resources when they are in a strong network position. When an individual is more network-centric, he or she will have more access to different types of knowledge and information, and the distribution and configuration of network resources are also faster. Therefore, this paper selects network centrality as the indicator of relationship network structure and examines its impact on stakeholder engagement behavior.

#### 2.1.2. Theory of Planned Behavior

The theory of planned behavior (TPB) is derived from the theory of reasoned action (TRA), which was developed by Fishbein [37], who argued that individual behavior is influenced by the individuals’ behavioral willingness, which in turn is determined by their attitudes and subjective norms. Ajzen [38] presented the TPB. He added perceived behavioral control to the TRA and considered that behavioral willingness is mainly determined by a combination of attitudes, subjective norms and perceived behavioral control. The TPB is mainly used to predict individual behavior and behavioral intentions. Behavioral attitude refers to the individual evaluation of how much he or she likes or dislikes a certain behavior. The more positive the behavioral attitude, the higher the behavioral willingness. Subjective norms are the social pressures that individuals feel when performing a behavior. The TPB suggests that these social pressures arise mainly from the attitudes and opinions of other important people. The social nature of people dictates the inevitable existence of subjective norms. Perceived behavioral control is the perceived ease of performing a behavior of an individual. When the individual perceives that the behavior can be performed competently, the behavioral willingness increases accordingly.

**Figure 2:**
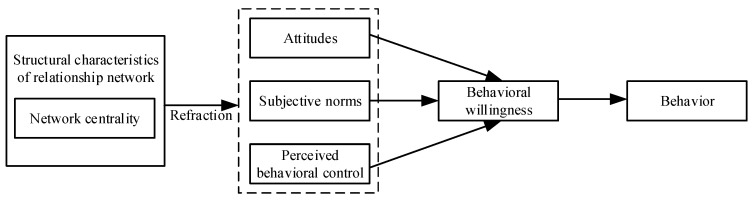
The TPB, incorporating network centrality.

The TPB has been widely used in various research studies. For example, Bamberg et al. [39], Moura et al. [40], Yadav and Pathak [41] and Barone [42] studied travel mode choice, water conservation behavior, green consumption behavior and food waste behavior, respectively, based on the TPB. In addition, the TPB is highly flexible and malleable, and it is also of theoretical importance to explore the ways it can be optimized or integrated. This paper considers the psychological activity of stakeholders’ behavior refracted through network centrality as a collection of attitudes, subjective norms and perceived behavioral control based on the TPB. The improved TPB is shown in Figure 2.

#### 2.1.3. Attitude–Context–Behavior Theory

Smith [43] proposed an intrinsic external-factor environment model based on a large number of experiments. The model suggests that individual behavior is a product of the interaction between the individual and the environment (economy, technology, culture, institutions). Based on Smith’s research, Guagnano et al. [44] proposed the attitude–context–behavior (ACB) theory, which suggests that individual behavior is not only influenced by attitudes but that the context also has an impact on the occurrence of the individual’s behavior. Individual behavior is the result of the interaction between attitude and context.

When attitudes and context are oriented in the same direction, pro-environmental behaviors will often occur, if individuals have positive pro-environmental attitudes and the external context is conducive to the occurrence of the behavior. Conversely, if individuals have negative pro-environmental attitudes and the external context is not conducive to the behavior, pro-environmental behavior will be severely hindered. When attitudes and contexts are oriented in opposite directions, the emergence of pro-environmental behavior depends on the strength of the influence. The ACB theory highlights the influence of external environmental factors on individual behavior. It provides the theoretical support for introducing the involvement climate into the theoretical influence model of the stakeholder relationship network structure on engagement behavior.

### 2.2. Research Hypothesis

Stakeholder engagement plays a crucial role in environmental and land management. Similarly, stakeholder engagement is considered to be one of the key factors for the success of BR. During the engagement process, stakeholder behaviors are motivated by emotions, perceptions, role interactions and relationships. According to network embeddedness theory, stakeholders’ engagement behavior and their behavioral willingness are influenced by the structure of social networks. In this paper, social network structure refers to the relationship network structure formed by the interweaving of contracts, cooperation, trust and other relationships among stakeholders. Since network centrality can better reflect the position, status and social prestige of stakeholders, in this paper it is used as a scale to measure the structural characteristics of relational networks. According to the findings of the TPB, stakeholder engagement behavior is influenced by behavioral willingness. Behavioral willingness refers to the psychological inclination and behavioral motivation for engaging in BR. According to ACB theory, the individual’s behavior is not only influenced by their own attitudes but also depends on the external environment. Involvement climate as an external environment also influences stakeholders’ engagement behavior. In this paper, the involvement climate is considered to be the importance and necessity of BR, as perceived by the whole organization.

**Figure 3:**
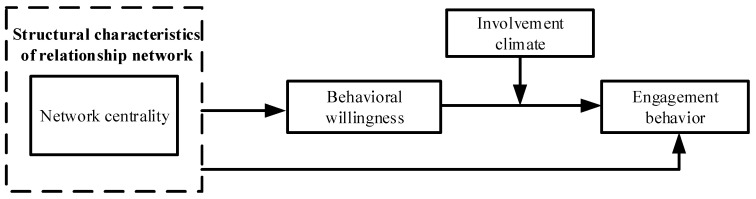
A theoretical model of stakeholder engagement behavior occurrence from the perspective of network embeddedness.

Thus, based on theories such as network embeddedness, TPB and ACB, and combined with the existing literature, this study selected network centrality as the antecedent variable, behavioral willingness as the intermediary variable and involvement climate as the moderating variable. A theoretical model of the impact of the relationship network structure on the engagement behavior of stakeholders was constructed (see Figure 3).

Researchers have found that individual engagement is influenced by social networks in the organization [27,45,46,47]. Specifically, BR is a social group activity. The nature of group activities and the high frequency of interactions dictate that the engagement behavior of BR stakeholders is influenced by relationship networks. The network centrality, as one of the key indicators of the social network structure, has received more attention from scholars [48,49,50,51]. Centrality reflects the degree to which an individual is at the center of the network and can represent the degree to which an individual is popular in the network. Network centrality can lead to greater control over resources, information and other social connections. Therefore, it is a good measure of an individual’s position, status and social prestige. Measures of network centrality include degree centrality, closeness centrality and betweenness centrality [52]. Degree centrality measures the amount of control over individual information resources, and individuals with a higher degree of centrality can gain more influence and more social support. Closeness centrality is the degree of centrality calculated as the shortest distance between two individuals. Betweenness centrality measures an individual’s ability to mediate information. Stakeholders with higher degree, closeness and betweenness centrality have a higher reputation and status in the relationship network, which in turn makes them show better engagement behavior in BR. Based on this, we propose the following hypotheses:

**Hypothesis** **1** **(H1).** *There is a positive impact of degree centrality on the engagement behavior of stakeholders*.

**Hypothesis** **2** **(H2).** *There is a positive impact of closeness centrality on the engagement behavior of stakeholders*.

**Hypothesis** **3** **(H3).** *There is a positive impact of betweenness centrality on the engagement behavior of stakeholders*.

Behavioral willingness refers to the psychological tendency of an individual to engage in a certain behavior. It reflects the subjective willingness of individuals. According to social network research, BR stakeholders with higher network centrality gain higher network influence. These network influences stimulate the stakeholders’ willingness to share knowledge and information, while high network influence is also conducive to mobilizing other stakeholders to engage in BR. Therefore, the subjective feelings and expectations of stakeholders with higher network centrality enhance the occurrence of engagement behaviors. In addition, some research scholars have studied the influence of social network structure on behavioral willingness. Ma et.al [53] found that social networks have a significant effect on individuals’ willingness to share knowledge in the process of innovation. Huang and Wang [54] studied the adoption of pro-environmental behaviors by farmers in rural tourism sites and found that farmers at the center of the network, such as large planters and rural elites, showed better willingness to adopt pro-environmental behaviors such as soil testing and fertilization.

According to the psychology–behavior conception, individual behavior is a concrete manifestation of behavioral willingness. The behavioral willingness contributes positively to the generation of individual behavior. Through empirical studies, Ma et al. [55] confirmed that the public’s behavioral willingness has a positive influence on the generation of its behavior. Lu et al. [56] found that the public’s willingness to save energy positively influenced energy-saving behaviors. Shi et al. [57] found that behavioral willingness played the same role in the occurrence of behaviors through studying the public’s behavioral willingness to use urban transport sharing products. The same is true for stakeholder engagement behavior in BR. The stronger the willingness of stakeholders to engage, the more likely they are to take actual actions to engage in BR.

The TPB suggests that behavioral willingness is the main antecedent of behavior. The behavioral willingness mediates the effects of attitudes, subjective norms and perceived behavioral control on behavior. Based on a modified TPB, Su et al. [58] found a significant intermediary effect of consumers’ behavioral willingness on the influence of consumers’ behavioral attitudes, subjective norms and perceived quality of competence on their engagement behavior. Wang and Yang [59] found that the logic of farmers’ engagement behavior adjustment in agricultural land rehabilitation followed the path of “perception → willingness → behavior”, and there was a complete intermediary effect of farmers’ behavioral willingness between perceptions and behavioral responses. Therefore, based on the TPB, in this paper, we consider the psychological expectations of stakeholders resulting from network centrality as a collection of attitudes, subjective norms and perceived behavioral control. The proposed hypotheses are as follows:

**Hypothesis** **4** **(H4).** *There is a positive impact of degree centrality on the behavioral willingness of stakeholders*.

**Hypothesis** **5** **(H5).** *There is a positive impact of closeness centrality on the behavioral willingness of stakeholders*.

**Hypothesis** **6** **(H6).** *There is a positive impact of betweenness centrality on the behavioral willingness of stakeholders*.

**Hypothesis** **7** **(H7).** *There is a positive impact of the stakeholders’ behavioral willingness on engagement behavior*.

**Hypothesis** **8** **(H8).** *The stakeholder behavioral willingness has an intermediary role between the network centrality and engagement behavior*.

ACB theory suggests that individual behavior is not only determined by attitudes towards behavior but is also influenced by the external environment. Green and Kreuter [60] proposed the antecedent–conduct behavior model, which considered the surrounding environment as a reinforcing factor for behavior. Climate has received increasing attention from scholars as one of the important environmental factors that influence the occurrence of behavior. Kahn [61] found that a good employee involvement climate could contribute to positive employee behavior at work. Higgins [62] concluded that the employee engagement climate promoted self-motivated rewards and job fulfillment among employees, mediated the match between the work environment and employee aspirations and helped to make employees feel fully present at work. Richardson and Vandenberg. [63] suggested that if an organization had a good employee involvement climate, it not only gave employees a good cognitive understanding of the workplace and its content but also gave them more freedom to work. He et al. [64] constructed a theoretical model of typical non-green consumption behavior and showed that the luxury and wastefulness ethos, as a manifestation of climate, had a significant moderating effect on the influence of face consciousness on typical non-green consumption behavior. Qin and Zhang [65] found that involvement climate was a very important contextual factor that has a positive moderating effect on the relationship between promotion orientation and individual innovation. The proposed hypothesis is as follows:

**Hypothesis** **9** **(H9).** *Involvement climate moderates the relationship between behavioral willingness and the engagement behavior of stakeholders in BR*.

## 3. Research Methodology

### 3.1. Stakeholder Identification

Considering the specificity of BR, this paper adopts the stakeholder definition of Freeman, that is, “any group or individual who can affect or is affected by the achievement of the organization’s objectives” [66]. Thus, stakeholders in BR are identified as those with roles involved in the recycling and pollution treatment phase stages. Currently, scholars mainly use methods such as brainstorming, Delphi or snowball sampling to identify stakeholders. A widely used technique for identifying stakeholders is snowball sampling [67]. This tool requires asking one actor to name other actors with the same or related stakes. These actors, in turn, are interviewed and asked to supply further names of interested actors. The process is repeated until the names start to repeat [68]. This study used snowball sampling to identify the stakeholders in BR in China, and we carried out seven semi-structured interviews with seven specialized actors in late 2017 and early 2018. The first interviewee was a professor in the research and technology units involved and a member of ENGOs. The second interviewee, in the government, played a key role in BR in China. The other four respondents included two former users and two consultancies. The results of the stakeholder identification process included governments, government departments, former users, pollution control companies, the public, consultancies, ENGOs, end users, news media and research and technology units.

### 3.2. Variables Measurement

The measurement of engagement behavior, behavioral willingness and involvement climate was conducted by referring to the existing scales of relevant scholars (Table 1), combining the actual situation of BR in China and expert recommendations. These measurements used a five-point Likert scale. The measurement of the relationship network structure was derived from the network data and was not part of the scale measurements.

The relationship network structure of stakeholders, engagement behavior, behavioral willingness, involvement climate and control variables were specifically measured as follows.

#### 3.2.1. The Measurement of Relationship Network Structure

A relationship network is made up of three components: nodes, lines and relationship strengths. In this paper, stakeholders such as government, government departments, former users and contamination treatment companies were used as relationship network nodes. Contractual, cooperative, communication, trust and incentive relationships between stakeholders were used as network relationships. Each line in the graph was assigned a certain value based on the strength of the connection between the two nodes, for example, strong ties between two stakeholders were assigned a value of 3, medium ties were assigned a value of 2 and no ties were assigned a value of 0. In this paper, the stakeholder relationship network graph was considered to be undirected, and its relationship matrix was symmetrical. The relationship matrix was entered into the UCINET 6 software for SNA, and the NetDraw function was used to transform the stakeholder relationship matrix into a visualized relationship network graph. Based on SNA, the network centrality included three metrics: degree centrality, closeness centrality and betweenness centrality.

(1) Degree centrality. Degree centrality is a relatively simple centrality metric, which is divided into absolute degree centrality and relative centrality. The absolute degree centrality is the number of other nodes that are directly connected to a node. If a node is directly connected to many other nodes, then it has a high absolute degree centrality. Supposing there are *M* nodes in a social network, and the maximum possible degree of any one node is *M* − 1. If the absolute degree centrality of a node is *d*(*n_i_*), then the calculation formula for the relative degree centrality is as follows:

(1) $$C_{D}^{\prime }\left(n_{i}\right)=\frac{d\left(n_{i}\right)}{M-1}$$

The relative degree centrality values are in the range [0, 1]. The higher the value, the greater the influence and the easier it is to obtain sufficient information resources.

(2) Closeness centrality. The closeness centrality of a node is a measure of the extent to which the node is not controlled by other nodes. A node is considered to have a high proximity centrality if its distance to all other nodes in the network is short. Closeness centrality includes absolute closeness centrality and relative closeness centrality. Absolute proximity centrality is the sum of the shortcut distances between a node and the other nodes in the network and is calculated as follows:

(2) $$C_{AMi}^{-1}=\sum_{j=1}^{n}d_{ij}$$

The relative closeness centrality is calculated as follows:

(3) $$C_{C}^{\prime }\left(n_{i}\right)=\frac{M-1}{\left[\sum_{j=1}^{N}d\left(n_{i},n_{j}\right)\right]}$$

The relative closeness centrality values are in the range [0, 1]. The larger the value, the closer the relationship between one stakeholder and other stakeholders and the easier the transmission of information resources in the network.

(3) Betweenness centrality. In social networks, the interaction between two non-adjacent nodes often relies on other nodes for implementation. Betweenness centrality is a measure of the bridging role of nodes, including absolute betweenness centrality and relative betweenness centrality. The absolute intermediate centrality is calculated as:

(4) $$C_{ABi}^{}=\sum_{j}^{n}\sum_{k}^{n}b_{jk}\left(i\right)$$

The absolute intermediate centrality is calculated as:

(5) $$C_{RBi}=\frac{2C_{ABi}}{n^{2}-3n+2}$$

The relative intermediate centrality values are in the range [0, 1]. A node with 0 relative centrality has no control over other nodes and is at the edge of the network. If the relative centrality of a node is 1, it has full control over the other nodes and is at the core of the network with the greatest network power. If the relative centrality of individual nodes is high, the information resources will be monopolized, which will have a negative impact on the stability of the network.

#### 3.2.2. The Measurement of Engagement Behavior

The engagement behavior measurement for BR stakeholders mainly referred to the studies of Ennew and Bink, Nicholas et al., Rasoolimanes et al. and Tosun. The measurement questions related to engagement behavior included “I engage in the tasks related to BR”, “I actively propose my needs”, “I often provide assistance to stakeholders associated with me”, “I provide suggestions to relevant stakeholders” and “I actively engage in meetings, work reports, surveys organized during the regeneration process”.

#### 3.2.3. The Measurement of Behavioral Willingness

The behavioral willingness measurement for BR stakeholders mainly referred to the studies of Ma et al. The measurement questions of behavioral willingness included “in general, I would like to engage in BR”, “I would like actively to engage in activities related to BR”, “I would like to collect and learn information resources of BR” and “I would like to mobilize people around me to engage in BR”.

#### 3.2.4. The Measurement of Involvement Climate

The involvement climate measurement for BR stakeholders was mainly based on the study of Richardson and Vandenberg, focusing on the employee involvement climate, with specific questions including “I feel that the decision-making on BR is democratic and fair”, “I feel that there are numerous training and education activities related to BR”, “I feel that the delivery and sharing of information resources related to BR is very timely and effective” and “I feel that incentives, and rewards are common in the process of BR”.

#### 3.2.5. The Measurement of Control Variables

In this paper, the years of experience and education levels of BR stakeholders were used for the measurement of control variables. The measurement questions included “what is the number of years you have engaged in BR” and “what is your highest academic background”.

### 3.3. Data Collection

Most BR in China is currently at the stage of recycling and contamination treatment. In this study, we selected the recycling and contamination treatment stage of BR of ten cities in China as the research object. Questionnaires were distributed in two forms: on-site distribution and email distribution. A total of 301 questionnaires were distributed, of which 245 were valid questionnaires (the response rate was 81.40%). The survey sample covered stakeholders such as governments, government departments, former users, pollution control companies, the public, consultancies, ENGOs, end users, news media and research and technology units. Those with less than 1 year of work experience accounted for 19.1%, with 36.1% having 1–3 years, 28.4% having 3–5 years, 10.8% having 5–10 years and 5.6% having 10 years or more. The proportions of academic qualifications were 37.9% with less than a bachelor’s degree, 40.2% with a bachelor’s degree and 21.9% with a postgraduate degree and above.

**Figure 4:**
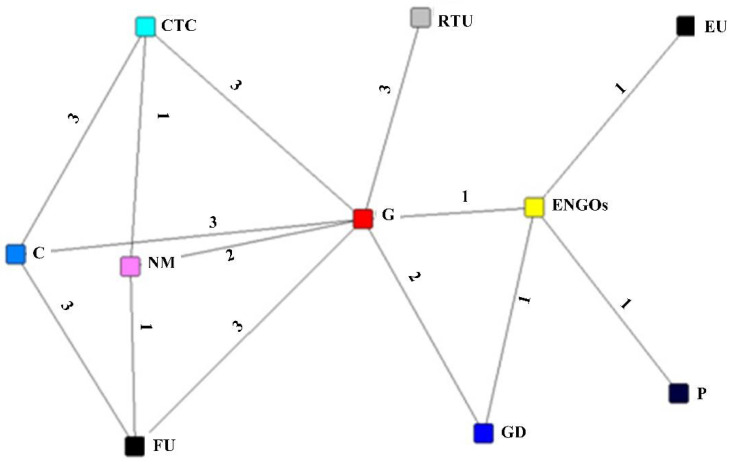
Relationship network of BR stakeholders at the recycling and contamination treatment stage.

The results of the questionnaire were used to deliberate and discuss the existence and strength of links between the various stakeholders in BR, resulting in a BR stakeholder relationship matrix, as shown in Table 2. The stakeholder relationship matrix can be transformed into a visual relationship network model using NetDraw. The stakeholder relationship network is shown in Figure 4, where the different nodes represent different stakeholders. Stakeholder degree centrality, closeness centrality and betweenness centrality were calculated using UCINET 6. The calculation process refers to social network analysis methods [73].

## 4. Results

### 4.1. Reliability and Validity Tests of Data

We used SPSS 21.0 to test the reliability and validity of the data, and the results are shown in Table 3. The cross-loadings between variables are shown in Table 4. The Cronbach’s alpha coefficient for each variable was greater than 0.750, implying that the questionnaire has good reliability. The AVE values for each variable in the test for convergent validity were all greater than 0.650, and the factor loadings for each variable were all greater than 0.700, indicating good convergent validity for each variable. The square roots of the AVE values for each variable were greater than the correlation coefficient with the other variables, indicating good discriminant validity between the variables.

### 4.2. Descriptive Statistical Analysis

The descriptive statistics for each variable are presented in Table 5. The mean values of the behavioral willingness, involvement climate and engagement behavior were 3.217, 2.872 and 3.004, respectively, indicating that the overall climate of engagement in BR was low and the level of stakeholder engagement behavior was not high. The degree centrality, closeness centrality and betweenness centrality were calculated using UCINET 6. They are not part of the scale measures and will not be analyzed in detail.

### 4.3. Common Method Bias

Common method bias is a systematic bias that affects the results to some extent. In this study, different methods were used to measure the variables, to reduce the common method bias. Stakeholder relationship networks were constructed using data from stakeholder relationship interactions, and the network centrality variables network was calculated using UCINET 6. The behavioral willingness, involvement climate and engagement behavior were measured using questionnaires. In this study, we used process control and statistical tests to control the common variance in the questionnaire data. In order to control the biases arising from process control, we ensured that the items were clearly stated, some ambiguous terms were removed and each stakeholder participated in the questionnaire independently during the data collection process. We used the Harman one-way test to examine the common methods. When not rotated, the variance contribution of the first factor was 38.42%, which is less than 40%. Overall, the problem of shared variance was not serious and had only a small impact on the results of the study.

### 4.4. Correlation Analysis

When one variable changes in response to another variable, this indicates that there is a correlation between the two variables. The change can be either a positive or negative covariance. Correlation analysis is generally used to analyze the closeness of the relationship between two variables. In this study, a Pearson correlation analysis was conducted using SPSS 21.0 to analyze the network centrality, behavioral willingness, involvement climate and engagement behavior, as shown in Table 6. It can be seen that all variables were significantly correlated at the 0.05 or 0.01 levels, meeting the basic requirements for the regression analysis of data. For example, degree centrality, closeness centrality and betweenness centrality were all significantly correlated with engagement behavior, with correlation coefficients of 0.653, 0.689 and 0.510. Behavioral willingness was also significantly correlated with engagement behavior, with a correlation coefficient of 0.945, and degree centrality, closeness centrality and betweenness centrality were also significantly correlated with behavioral willingness, with correlation coefficients of 0.654, 0.709 and 0.541.

### 4.5. Regression Analysis

A hierarchical regression analysis was conducted using SPSS 21.0 to test the relevant hypotheses. The main effects of degree centrality, closeness centrality and betweenness centrality on engagement behavior, the intermediary effect of behavioral willingness and the moderating effect of involvement climate were tested.

#### 4.5.1. Main Effects Test

In order to test the main effects of degree centrality, closeness centrality and betweenness centrality on the engagement behavior, models 1, 2, 3 and 4 were constructed with engagement behavior as the dependent variable. Model 1 included only control variables such as the years of work and academic qualifications. Model 2, model 3 and model 4 were formed by adding the degree centrality, closeness centrality and betweenness centrality variables to model 1. As can be seen from Table 7, the control variables explained only 2.174% of the engagement behavior variance in model 1. After adding the degree centrality, closeness centrality and betweenness centrality into model 1, 17.13%, 14.052% and 10.961%, respectively, of the variance in engagement behavior was explained. The regression coefficients of the three variables were 0. 251, 0. 460 and 0.004. These findings imply that degree centrality, closeness centrality and betweenness centrality all have a significant positive effect on engagement behavior. Therefore, hypotheses H1, H2 and H3 were tested.

#### 4.5.2. Intermediary Effect Test

Type I error rates and statistical test power are traditional tests for intermediary effects [74], but the level of testing is relatively weak. In this study, we adopted the intermediary effect test step of Wen [75] and the bootstrap method of testing the mediating effect proposed by Shrout et al. [76], which are commonly used by scholars nowadays. The bootstrap method is not restricted to a positive terrestrial distribution. To investigate the important role of indirect effects, the bias-corrected confidence intervals generated by the bootstrap method were used. The 2.5th percentile and 97.5th percentile values were taken as upper and lower bounds to check whether 0 is present in the 95% CI. This determines whether indirect effects are present, as shown in Table 8.

From Table 8, it can be seen that the total effect of behavioral willingness (β = 0.0256, *p* < 0.001; β = 0.0415, *p* < 0.001; β = 0.0284, *p* < 0.001) and the indirect effect (β = 0.0232, *p* < 0.001; β = 0.0392, *p* < 0.001; β = 0.0285, *p* < 0.001) were significant. This implies the intermediary effect of the behavioral willingness, and the hypotheses H4, H5, H6, H7 and H8 were tested. It is worth noting that the intermediary role of behavioral intention is far greater than that of degree centrality and betweenness centrality.

#### 4.5.3. Moderating Effects Test

If the relationship between an independent variable (X) and a dependent variable (Y) is moderated by another independent variable (R), then the variable R is the moderating variable. The direction of moderating variable influence can be positive or negative, and the strength of the influence can vary between strong and weak. The amount of change in the model’s goodness of fit (R2) is decisive in determining whether the moderating effect is significant. If ∆R2 is positive, the new model is better than the old one. Therefore, the larger the ΔR2 value obtained by introducing the interaction term to the model, the more significant the moderating effect of the moderator variable.

**Figure 5:**
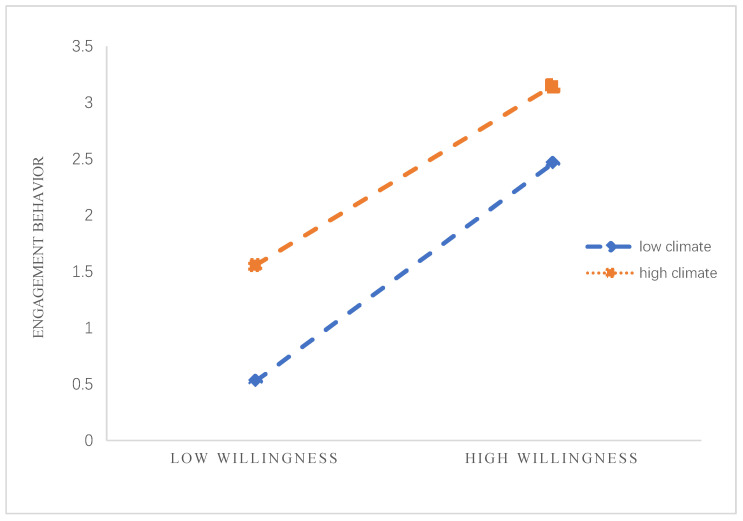
Moderating effect of involvement climate.

In order to test the moderating effect of involvement climate between behavioral willingness and engagement behavior, model 5 of the effect of behavioral willingness on engagement behavior was constructed, while model 6 was constructed by introducing involvement climate variables to model 5. Finally, model 7 was constructed by using PROCESS in SPSS 21.0. The F value of model 7 was 318.955, with *p* < 0.001 and ∆R2 = 0.959, which is greater than 0 and also greater than the values for model 5 and model 6, as shown in Table 9. The regression coefficient of the interaction term between behavioral willingness and involvement climate was 0.354, and the participation atmosphere had a significant positive moderating effect on behavior intention and participation behavior path. Based on the study of Aiken et al. [77], the moderating effect diagram of involvement climate is shown in Figure 5. The slope of the regression line of the relationship between behavioral willingness and engagement behavior is greater for higher involvement climate conditions, implying a stronger positive effect of behavioral willingness on engagement behavior.

## 5. Discussion and Implications

### 5.1. Discussion and Implications of the Impact of Network Centrality on Engagement Behavior

The results show that the network centrality of BR stakeholders has a significant influence on behavioral willingness and engagement behavior. Stakeholders with higher degree centrality, closeness centrality and betweenness centrality have richer information resources, which largely contributes to the formation of higher engagement behaviors. In addition, stakeholders with higher network centrality can fully mobilize the behavioral willingness and engagement behavior of other stakeholders. The engagement behavior is enhanced in terms of the stakeholder network centrality as follows:

(1) The government can enhance engagement behavior by consolidating their own network centrality. Specifically, in the recycling and pollution treatment stage of BR, the government should focus on strengthening its own network relationships with other stakeholders, especially with ENGOs, the news media, the public and end users. As the initiator and governor of BR, the government should avoid the phenomenon of “lazy governance” and always put the health and safety of the public and end users at the forefront of BR. The government should also formulate social engagement policies, encourage other stakeholders to actively participate in BR and actively listen to the views of the public, end users, ENGOs and other stakeholders on these policies. Through news media, public opinion and the monitoring role of ENGOs, the government can defend its network-centric power and network influence. In this way, the government not only obtains a higher prestige but also to a certain extent improves the network status of the public and end users, making their own engagement behavior more altruistic.

(2) The public and end users can increase engagement behavior by increasing their own social influence. In the recycling and pollution treatment stage of BR, the public and end users are located at the periphery of the relationship network, having less frequent interaction with other stakeholders and less control over information resources. An increase in social influence directly leads to an increase in their network centrality. By increasing their social influence, the public and end users can gain access to more network information and knowledge resources, thereby improving their position in the relationship network accordingly and increasing their initiative and the efficiency of their engagement behavior. For example, the public and end users should strengthen their ties with the news media. Members of the public and end users who have made outstanding contributions to the task of BR can be publicized through the promotion of typical figures by the news media, thereby increasing the social influence of the public and end users.

(3) ENGOs and news media should enhance their engagement by full use of their monitoring function. As third parties in BR, ENGOs and the news media should strengthen their links with the government, the public and end users, and effectively safeguard their own monitoring rights. Specifically, ENGOs should consider protecting the interests of the public and end users as their responsibility and should communicate with the government promptly and educate the public and end users about BR from time to time. The news media should follow up and publicize BR-related information in a timely manner and evaluate BR events objectively and fairly, which not only protects their monitoring rights but also encourages the public and end users to engage in BR and contribute to the success of BR.

### 5.2. Discussion and Implications of the Intermediary Role of Behavioral Willingness

Stakeholders with higher degree centrality, closeness centrality and betweenness centrality have richer information resources, resulting in a higher awareness of the economic, ecological and social benefits provided by BR and a positive willingness to engage in BR. Behavioral willingness also has a significant positive influence on engagement behavior. The higher the behavioral willingness of stakeholders, the stronger their perceptions of stakeholder engagement at the psychological level. Therefore, they are willing to share information and resources, and mobilize other stakeholders to effectively engage in BR, thus promoting better engagement behavior.

Based on the intermediary effect results for behavioral willingness, stakeholders have higher network centrality, which can effectively improve behavioral willingness and thus promote positive engagement behavior. The engagement behavior is enhanced in terms of the stakeholder behavioral willingness as follows:

(1) The government enhances engagement behavior by raising their own awareness and leading decision-making. As the main decision-maker, the government should always be aware that it is the main organization in charge of BR and should strengthen its awareness of leadership in decision-making. Specifically, the government should keep abreast of the actual situation regarding BR and understand the willingness of other stakeholders to engage, in order to scientifically formulate and adjust the relevant policies and implementation plans for BR. For example, it should build an engagement mechanism and engagement platforms for shared governance among different stakeholders. These measures could raise the engagement enthusiasm of the public, end users and third parties, effectively fulfill the leading decision-making responsibility of the government and transform its decision-making participation awareness into active participation behavior.

(2) The public, end users and third parties should increase their engagement behavior by raising their own awareness of co-engagement. The public, end users and third parties should respond positively to the scope, means and mechanism of engagement, for example, material and spiritual rewards for engagement, co-engagement platforms and online co-engagement APPs set by the government. They should actively join ecological and environmental voluntary service organizations or teams, to form voluntary service organizations with distinctive themes and effective social influence, and should contribute to the sustainability of BR by increasing their enthusiasm for engagement.

### 5.3. Discussion and Implications of the Moderating Role of Involvement Climate

The involvement climate plays a moderating role between behavioral willingness and engagement behaviors. When stakeholders perceive a climate of democratic and fair decision-making regarding BR, diverse BR training, publicity and education activities, positive incentive policies and rewards and effective information resource sharing, their behavioral willingness will be subsequently increased and a relatively positive engagement behavior will be shown.

As the central decision-maker in participatory decision-making, the government should pay attention to the moderating effect of the involvement climate and create a climate of democratic and fair engagement in BR. When making decisions on BR, the government should ensure that the decision-making process is democratic and fair and that all stakeholders are able to engage efficiently in BR. Stakeholders such as the government, government departments and ENGOs should share their knowledge on BR with the public and end users in a timely manner and organize talks on BR regularly. For example, the government and government departments could invite brownfield researchers from universities or other scientific research and technology units to give lectures on the topics of “brownfield sites and brownfield regeneration” and “sustainable brownfield regeneration design”. A diverse publicity atmosphere for BR should be created. On the basis of the existing brownfield regeneration publicity, the news-related publicity for BR should be promoted and improved. In order to adapt to audience-oriented and differentiated communication trends, various types of new media publicity products for BR could be developed, such as increasing the development of short video products. In addition, innovative social promotion of BR could be undertaken. Combined with themes such as World Environment Day and National Low-Carbon Day, various forms of environmental protection publicity activities could be organized to guide people to consciously participate in environmental protection activities.

## 6. Conclusions and Future Directions

This paper focused on the influence of the relationship network structure on engagement behavior, expanding the application of social network theory and also helping to enrich stakeholder research. Stakeholder engagement in BR is an economic and social behavior embedded in the relationship network structure. In this paper, we constructed a theoretical model of stakeholder engagement behavior generation based on network embeddedness theory, the theory of planned behavior and ACB theory. The results showed that degree centrality, closeness centrality and betweenness centrality have a positive influence on the engagement behavior of stakeholders. Behavioral willingness has a significant intermediary effect between network centrality and engagement behavior. The involvement climate moderates the relationship between behavior willingness and engagement behavior.

Considering the current situation regarding BR in China, this study took BR in ten cities of China as an example and investigated the influence of network centrality in the stakeholders’ relationship network structure on engagement behavior at the recycling and contamination treatment stages. However, the relationship networks at different stages and other indicators of the relationship network structure may have different impacts on the engagement behavior of stakeholders. Therefore, subsequent studies could focus on the influence of different relationship network structure indicators on engagement behavior at other stages of BR, to further extend the research.

## Figures and Tables

**Table 1:** Measurement questions and sources of scales.

Variables	Reference Scale
Engagement behavior	Ennew and Bink [69], Tosun [70], Nicholas et al. [71], Rasoolimanes et al. [72]
Behavioral willingness	Ma et al. [55]
Involvement climate	Richardson and Vandenberg [63]

**Table 2:** Relationship matrix of BR stakeholders at the recycling and contamination treatment stage.

Stakeholders	Government	Government Departments	Former Users	Contamination Treatment Companies	Public	Consultancies	Environmental NGOs	End Users	Research and Technology Units	News Media
Government	0	2	3	3	0	3	1	0	3	2.328
Government Departments	2	0	0	0	0	0	1	0	0	0
Former users	3	0	0	0	0	3	0	0	0	0.887
Contamination treatment companies	3	0	0	0	0	3	0	0	0	0.904
Public	0	0	0	0	0	0	1	0	0	0
Consultancies	3	0	3	3	0	0	0	0	0	0
ENGOs	1	1	0	0	1	0	0	1	0	0
End users	0	0	0	0	0	0	1	0	0	0
Research and technology units	3	0	0	0	0	0	0	0	0	0
News media	2	0	1	1	0	0	0	0	0	0

**Table 3:** Results of reliability and validity tests of the scales.

Variables	α	CR	AVE	Behavioral Willingness	Involvement Climate	Engagement Behavior	Degree Centrality	Closeness Centrality	Betweenness Centrality
Behavioral willingness	0.830	0.887	0.664	0.814					
Involvement climate	0.818	0.889	0.668	0.596	0.817				
Engagement behavior	0.963	0.971	0.874	0.749	0.799	0.934			
Degree centrality				0.654	0.647	0.653	1		
Closeness centrality				0.709	0.692	0.689	0.930	1	
Betweenness centrality				0.541	0.490	0.510	0.708	0.699	1

**Table 4:** Results for the factor loadings corresponding to each question item.

Title Item	Factor 1	Factor 2	Factor 3	Degree Centrality	Closeness Centrality	Betweenness Centrality
In general, I would like to engage in BR	0.845					
I would like actively to engage in activities related to BR	0.823					
I would like to collect and learn information resources of BR	0.845					
I would like to mobilize people around me to engage in BR	0.743					
I feel that the decision-making of BR is democratic and fair		0.814				
I feel that there are numerous training and education activities related to BR		0.869				
I feel that the delivery and sharing of information resources related to BR is very timely and effective		0.868				
I feel that incentives, and rewards are common in the process of BR		0.707				
I engage in the tasks related to BR			0.961			
I actively propose my needs			0.942			
I often provide assistance to stakeholders associated with me			0.950			
I provide suggestions to relevant stakeholders			0.943			
I actively engage in meetings, work reports, surveys organized during the regeneration process			0.876			
Degree centrality				1		
Closeness centrality					1	
Betweenness centrality						1

**Table 5:** Results of descriptive statistical analysis.

Variables	Min.	Median	Max.	Average Value	Marking Deviation
Degree centrality	9.091	36.364	100.000	36.068	23.290
Closeness centrality	31.429	55.000	100.000	54.515	15.146
Betweennes centrality	0.000	2.333	68.788	8.762	16.416
Behavioral willingness	2.250	3.250	4.750	3.217	0.479
Involvement climate	1.750	3.000	4.250	2.872	0.532
Engagement behavior	1.200	3.400	5.000	3.004	0.913

**Table 6:** Correlation analysis of variables.

Variables	Working Years	Education Level	Degree Centrality	Closeness Centrality	Betweenness Centrality	Behavioral Willingness	Involvement Climate	Engagement Behavior
Working years	1	0.128						
Education level	0.186	1						
Degree centrality	0.268	0.117	1					
Closeness centrality	0.211	0.139	0.930 **	1				
Betweenness centrality	0.154	0.152	0.708 **	0.699 **	1			
Behavioral willingness	0.383 *	0.459 **	0.654 **	0.709 **	0.541 **	1		
Involvement climate	0.352 *	0.399 **	0.647 **	0.692 **	0.490 **	0.937 **	1	
Engagement behavior	0.359 *	0.474 **	0.653 **	0.689 **	0.510 **	0.945 **	0.960 **	1

Note: ** indicates significance at the *p* < 0.01 level, * indicates significance at the *p* < 0.05 level.

**Table 7:** Main effects test of variables.

Variables	Engagement Behavior			
	Model 1	Model 2	Model 3	Model 4
Working years	0.235	0.242	0.226	0.205
Education level	0.180	0.257	0.235	0.219
Degree centrality		0.684 ***	0.253 ***	0.251 ***
Closeness centrality			0.461 ***	0.460 ***
Betweenness centrality				0.004 ***
R^2^	0.094	0.556	0.584	0.584
Adjusted R2	0.051	0.524	0.543	0.531
F	2.174	17.13	14.052	10.961

Note: *** indicates significance at the *p* < 0.01 level.

**Table 8:** Test for intermediary effects of behavioral willingness.

Category	Estimated Value	SE	t	Bootstrapping	
Degree Centrality–Engagement Behavior				LLCI	ULCI
Total effect	0.0256	0.0045	5.6523	0.0165	0.0347
Direct effect	0.0024	0.0026	0.9390	−0.0028	0.0077
Indirect effect	0.0232	0.0054		0.0135	0.00345
**Category**	**Estimated value**	**SE**	**t**	**Bootstrapping**	
**Closeness Centrality–Engagement Behavior**				**LLCI**	**ULCI**
Total effect	0.0415	0.0067	6.2263	0.0281	0.550
Direct effect	0.0023	0.0043	0.5318	−0.0068	0.067
Indirect effect	0.0392	0.0086		0.0246	0.0576
**Category**	**Estimated value**	**SE**	**t**	**Bootstrapping**	
**Betweenness Centrality–Engagement Behavior**				**LLCI**	**ULCI**
Total effect	0.0284	0.0045	5.6523	0.0137	0.0431
Direct effect	0.001	0.0034	0.0222	−0.0068	0.0067
Indirect effect	0.0285	0.0113		0.0130	0.0549

**Table 9:** Test for moderating effect of involvement climate.

Variables	Engagement Behavior		
	Model 5	Model 6	Model 7
Behavioral willingness	0.945 ***	0.365 **	2.018 ***
Involvement climate		0.618 ***	1.991 ***
Behavioral willingness × Involvement climate			0.354 ***
R^2^	0.892	0.939	0.979
Adjusted R^2^	0.890	0.936	0.959
F	355.48	321.329	318.955

Note: *** indicates significance at the *p* < 0.01 level, ** indicates significance at the *p* < 0.01 level.

## Data Availability

Data sharing is not applicable to this article.
